# Psychosocial Intervention for Family Caregivers of ALS Patients: A Systematic Review

**DOI:** 10.3390/healthcare12121171

**Published:** 2024-06-10

**Authors:** Leah Katz, Ayelet Gur

**Affiliations:** 1 Social Work Department, Tel Hai College, Qiryat Shemona 1220800, Israel; leakat@m.telhai.ac.il; 2 Research Center for Innovation in Social Work, Tel Hai College, Qiryat Shemona 1220800, Israel

**Keywords:** amyotrophic lateral sclerosis, ALS, family caregivers, psychosocial intervention, support program, systematic review

## Abstract

Proposal: This systematic review aims to comprehensively examine all existing knowledge on psychosocial interventions for family caregivers for ALS patients. Also, the study will present the gaps in knowledge, recommendations for future research, and guidelines for psychosocial interventions that are focused and adapted to the needs of family caregivers of ALS patients. Materials and methods: The systematic review was conducted according to the PRISMA guidelines and identified studies on psychosocial intervention for family caregivers of ALS patients, using five electronic databases: PsychNET, PubMed, EBSCO, PRIMO, and PROQUEST. Seven articles met the criteria and were included in the review. A thematic analysis was conducted to extract major themes. Results: Three major themes emerged from the data: (1) Personal benefits; (2) Interpersonal benefits; and (3) Charting challenges and pathways to improve psychosocial interventions. Conclusions: Based on the findings, practical guidelines were formulated that focus on the group’s composition, the facilitator’s role, the contents, the relationships within the group, and the opportunities and limitations of online interventions.

## 1. Introduction

Amyotrophic lateral sclerosis (ALS) is a progressive, neurodegenerative, and inevitably fatal disease associated with the loss of upper and lower motor neurons. There is no cure for ALS; life expectancy is usually 2–5 years after symptoms appear [1]. The diagnosis greatly affects both the lives of the patients and the lives of the caregivers.

The cognitive and behavioral impairments experienced by patients with ALS have wide-ranging consequences, impacting not only their own well-being but also significantly increasing the burden and anxiety felt by their caregivers [2,3]. The caregivers of individuals with ALS lead challenging lives, dedicating most of their time to caring for their loved ones and navigating complex situations, often at the expense of their own self-care [4]. This demanding caregiving role can persist for extended periods, creating prolonged stress and emotional strain [4]. Research indicates that higher levels of caregiver burden are associated with greater behavioral and physical impairment in patients and an increased likelihood of experiencing depressive symptoms [3].

Aoun et al. emphasized the predominance of caregiver burden and quality-of-life studies in the realm of motor neuron disease family caregivers. However, they propose a shift in focus toward the development of interventions that offer direct practical and psychosocial support for these caregivers [5]. There is a lack of studies on psychological interventions for people with ALS and their caregivers [6]. Although international awareness of the psychological burden and distress in ALS caregivers has increased and caregivers express a need for psychosocial support, supportive evidence-based interventions for these caregivers are lacking, and there is insufficient evidence available regarding specific treatments for family members caring for ALS patients [3,7].

Despite the limited research available on psychological interventions for people with ALS and their caregivers [6], there has been an increased international awareness of the psychological burden and distress experienced by ALS caregivers. Caregivers themselves express a clear need for psychosocial support [3,7]. However, the current body of evidence lacks supportive, evidence-based interventions specifically tailored to address the needs of ALS caregivers, and there is insufficient information on specific treatments for this population [3,7].

In light of these research gaps, conducting a systematic literature review becomes essential. Despite the limited existing knowledge, a systematic review allows for a comprehensive exploration of the available research on psychosocial interventions for family caregivers of ALS patients worldwide. The aim of this review is to identify effective interventions that are aligned with the unique needs of caregivers while also highlighting the areas where further research is required. By conducting a systematic review, we can bridge the gap between the existing knowledge and the necessary development of evidence-based interventions for ALS caregivers.

## 2. Method

### 2.1. Search Strategy

The protocol for this systematic review is registered with INPLASY under the registration number INPLASY202450084. In addition, the PRISMA guidelines were followed in conducting this systematic review [8]. The electronic databases EBSCO, PubMed, Primo, PSYNET, and PROQUEST were systematically searched using the following keywords along with synonyms. For each characteristic (amyotrophic lateral sclerosis, caregiver, intervention), we used multiple terms to enhance our ability to find as many relevant articles as possible. For the characteristic of ALS, we used the terms: “amyotrophic lateral sclerosis” and “ALS”. For the characteristic of caregiver, we used the terms “caregiver”, “caregiving”, “spouses”, “family members”, “husband”, “wife”, “couple”, “marriage”, “partner measure”, “significant others” and “dyad”. For the characteristic of intervention, we used the terms “intervention”, “group intervention”, “treatment”, “group psychotherapy”, “psychotherapy”, “individual psychotherapy”, “psychotherapeutic counseling”, “psychotherapeutic techniques”, “supportive psychotherapy”, “support groups” and “social support”. In conducting the screening process, it is important to note that no restrictions were placed on the publication years of the studies, ensuring a comprehensive inclusion of relevant investigations.

### 2.2. Selection Criteria

#### 2.2.1. Eligibility Criteria

The review incorporated both quantitative and qualitative research studies, encompassed studies concentrating on psychosocial interventions tailored to family caregivers of individuals diagnosed with ALS. No restrictions were imposed regarding the publication dates of the articles or the age demographics of the participants. 

#### 2.2.2. Exclusion Criteria

Studies were excluded if they did not focus on the psychosocial intervention for family caregivers of ALS patients. For example, the study by Kennedy et al. [9] was excluded because although it assesses psychological distress, quality of life, and burden in informal caregivers, it does not involve a psychosocial intervention. Even if the intervention was for the family caregivers, if the support was not focused on the caregivers and adapted to their needs to improve their quality of life, the study was excluded. For example, respite care and health services interventions were excluded. Systematic reviews were not included in this review.

### 2.3. Selection of Studies

**Figure 1:**
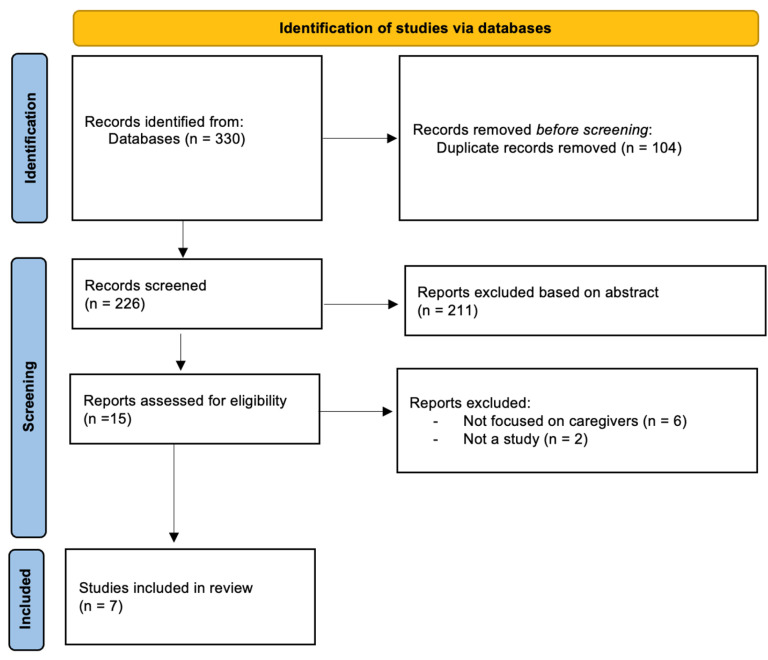
Review process of articles for inclusion in systematic review (PRISMA flowchart).

Studies were included and excluded by the inclusion and exclusion criteria. The database search identified a total of 330 possibly relevant articles. After eliminating duplicates, the total number of articles was reduced to 226. Subsequently, two authors independently examined the abstracts of these articles to ascertain their compliance with the inclusion criteria. Any disparities in judgment were deliberated upon, and a mutual agreement was achieved through discussion. After solving the conflict about the articles and reaching a consensus, all but 15 articles were excluded. A total of 6 studies were included in the review after reading the full-text screening (see Figure 1).

### 2.4. Date Coding

Articles were analyzed for content related to psychosocial intervention for family caregivers of ALS patients. Thematic analysis was used to extract major themes from the studies’ findings [10]. Both authors performed data analysis individually; in this process, we sorted codes and found potential themes. The second phase, in which themes were reviewed to understand what major stories appeared in the data, was completed jointly by the authors. In the final phase, the main themes were grouped to allow a clear and comprehensive presentation of the findings.

### 2.5. Rigorous Assessment

In evaluating the risk of bias, we employed a quality assessment approach utilizing the “standard quality assessment criteria for evaluating primary research papers from various fields”, as proposed by Kmet et al. [11]. This assessment methodology has been previously employed in a systematic review within the domain of caregivers of individuals with disabilities, as documented by Gur and Reich [12]. Each reviewed article adequately delineated its objectives, provided clear and appropriate study designs, and elucidated the contextual framework for the research. In five articles, the conclusions were substantiated by the results obtained, while in the remaining two articles, conclusions were only partially supported by the findings. The quantitative research endeavors undertook measures to control for confounding variables, and they provided estimates of variance for the primary results. The sample sizes were deemed appropriate, and the outcome and exposure measures were carefully defined and resilient to measurement or misclassification biases. Moreover, the assessment methods were disclosed, allowing for interventional approaches and random allocation where feasible. Participant characteristics were clearly outlined in four of the studies, while in the remaining three studies, participant characteristics were only partially reported. The qualitative studies established connections to a theoretical framework or broader knowledge base and elucidated relevant and justified sampling strategies. All five qualitative research endeavors provided clear descriptions of their data analysis procedures. Four out of the five studies clearly outlined systematic data collection methods, while one study provided only a partial description. Only one of the qualitative studies explicitly assessed the potential impact of the researcher’s personal characteristics and methodologies on the obtained data.

## 3. Results

### 3.1. Characteristics of Articles Included in the Review

Table 1 summarizes the main characteristics of six reviewed articles.

Study sample sizes ranged from 12 [4,13,14] to the largest sample size of 106 participants [7]. In two studies, the groups included caregivers of ALS patients and caregivers of PMA patients without separation [3,7], while the other studies focused on groups for caregivers of ALS patients only.

Study Locations: The studies were conducted in three European countries. Four were in Italy [4,6,14,15], one was in Denmark [13], and two were in the Netherlands [3,7].

Type of study: This review includes five qualitative studies [3,4,6,13,15] and two quantitative studies [7,14].

Publication Date Range: All articles were published between 2016 and 2022.

**Table 1:** Characteristics of articles included in the review.

	Authors	Country	Participants (Only ALS Caregivers)	Aim of Study	Study Design	Intervention
1	Bilenchi et al. [15]	Italy	N = 13 Gender: 8 females, 5 males Age: Mean age 61.38 years	To describe the implementation of a structured psychoeducational intervention in ALS by identifying the needs of both patients and their caregivers.	An explorative qualitative design	Psychoeducational groups for people with ALS and their caregivers.
2	Cipolletta et al. [4]	Italy	N = 12 Gender: Partners (spouse with ALS)—2 female, 4 males. Adult children (Parent with ALS)—5 females, 1 male. Age: Partners—Mean age 62 years (range 54–69). Adult children—Mean age 41.5 years (range 32–49).	To identify caregivers’ needs and experience as well as understand how this intervention strategy might help them. To explore differences between caregivers who are partners or children of patients with ALS.	Analysis of session transcripts and the semi-structured interviews conducted with the participants post-intervention.	Two mutual support groups for family caregivers of ALS patients.
3	de Wit et al. [3]	The Netherlands	N = 13 Not including caregivers of people with progressive muscular atrophy (PMA) Characteristics of ALS caregiver participants not specified separately from caregivers of people with PMA.	To gather insight into experiences with the different components of the program and to discover what caregivers gained from following the support program.	A randomized controlled trial	A blended psychosocial support program based on Acceptance and Commitment Therapy was developed to support partners of people with ALS and PMA. The intervention consisted of psychoeducation, psychological, and mindfulness exercises.
4	de Wit et al. [7]	The Netherlands	N = 106 Not including caregivers of people with PMA Characteristics of ALS caregiver participants not specified separately from caregivers of people with PMA. Intervention group: 52 Waitlist group: 52	To evaluate whether a blended psychosocial support program for caregivers of patients with ALS and PMA, aimed at enhancing the feeling of control over caregiving, reduces psychological distress.	A randomized controlled trial.	A blended psychosocial support program based on Acceptance and Commitment Therapy was developed to support partners of patients with ALS and PMA.
5	Marconi et al. [6]	Italy	Caregivers- (N = 18) Gender: Not specified. Mean age—57.8 years.	To investigate the experience of a meditation training program tailored for ALS needs to bring attention to the present moment and promote acceptance, eventually resulting in better quality of life.	Randomized clinical trial	Psychological intervention–meditation training for people with ALS and their caregivers.
6	Olesen et al. [13]	Denmark	N = 12 (3 male, 9 female) Age—18–67 Married/partner—11 Adult child—1	To evaluate the acceptability of a new online palliative rehabilitation blended learning program (EMBRACE) for family caregivers of people with ALS and cognitive and/or behavioral impairments.	Qualitative cross-sectional design	The EMBRACE intervention—an online palliative rehabilitation program for family caregivers—group meeting. EMBRACE had a blended learning format, combining both videos and virtual group meetings.
7	Sharbafshaaer et al. [14]	Italy	N = 12 The mean age of the caregivers was 57.81 years (ranging from 53.29 to 60.33 years). In the treated group (TG): 83.3% of the caregivers were spouses. 16.7% were sons/daughters of the patients. In the control group (CG): 71.4% of the caregivers were spouses. 28.6% were sons/daughters of the patients.	To explore the potential role of psychological support interventions for family caregivers of patients with ALS during the COVID-19 pandemic. Specifically, the study aimed to assess the impact of resilience-oriented group therapy sessions delivered via telemedicine on caregiver burden, resilience, and perceived stress.	A pilot, randomized, controlled trial	A resilience-oriented group therapy sessions delivered via telemedicine.

### 3.2. Interventions’ Characteristics

All studies presented group interventions that can be classified into three types: online intervention [13], face-to-face intervention [4,6,15], blended intervention with both components [3,7], and telemedicine intervention [14]. Of the articles that reported the number of meetings, the range was between 8 and 16 sessions.

Five interventions were structured with a protocol that included the topics of every session [3,6,13,14,15] compared to the others. Even though the facilitators coordinated the group sessions, they chose not to predetermine specific topics for each session. Instead, they decided to base the intervention on the group processes and adapt it to the specific topics and needs that arose spontaneously from the caregivers [4,7].

Two articles presented an intervention that included two groups for caregivers, one for partners and one for adult children [4,14], while the others presented heterogeneous groups: both caregivers and people with ALS [6,15], caregivers who include partners and adults children [13], and a group of partners who care for people with ALS and PMA [3,7]. Six articles present the familial relationship of the caregivers to the person with ALS [3,4,7,13,14,15], while one article did not specify the familial relationship to the person with ALS [6].

Six of the interventions were performed under the guidance of a psychologist [3,4,7,14,15], while one of them was part of the multidisciplinary team [15]. In two of these interventions, the psychologists went through training on how to conduct the intervention [3,7]. One intervention was conducted by healthcare professionals with years of experience working with ALS patients and their families [13]. In one study, two trainers conducted the intervention [6].

### 3.3. Theme 1: Personal Benefits

This theme encompasses contributions made in the personal domain, which can be further divided into two sub-themes: (1) psychological benefits, highlighting the impact of interventions on the psychological and emotional state of the participants, and (2) instrumental benefits, focusing on the contributions of the intervention in enhancing participants’ skills and effectiveness in parenting tasks.

- Psychological benefits

This sub-theme encompasses several positive psychological outcomes experienced by participants as a result of the intervention. These contributions include feelings of being acknowledged, taking a moment for oneself, gaining a sense of control and confidence, and fostering personal growth.

In terms of feeling acknowledged, caregivers expressed satisfaction in receiving dedicated attention, which made them feel listened to and empathized with. This recognition underscored the significance of their role within the caregiving process [3]. Everything around them usually concerned the person with ALS, so directing their focus from the person with ALS’s welfare to their needs was seen as a key advantage of the intervention, although they sometimes had to be reminded to focus on their own needs and challenges rather than those of the person with ALS [13]. In addition, interventions helped caregivers cope with emotions and thoughts and be able to accept and understand that it is common among caregivers of people with ALS to experiencing difficult situations [3,15].

Regarding the opportunity for caregivers to take a moment for themselves, participants expressed anticipation for the meetings, which they regarded as a welcomed respite from their daily routines filled with various responsibilities such as work, caregiving, and providing support for ALS patients [13]. Caregivers frequently struggled to prioritize self-care due to the substantial amount of time dedicated to meeting the needs of individuals with ALS [14]. Additionally, participation in group sessions facilitated a reduction in the “symbiotic” dynamic between the person with ALS and the caregiver, as it instilled a sense of assurance that caregivers were not solely responsible for managing all aspects of the situation [6].

In terms of cultivating a sense of control and confidence, caregivers reflected on their caregiving responsibilities both presently and in the future, as well as the division between their personal time and caregiving duties, as a result of the program [3]. Interventions also contributed to caregivers feeling more self-assured about their coping mechanisms [3,4,13]. Hearing the perspectives of others enabled caregivers to better comprehend the viewpoints of individuals with ALS and prompted them to reconsider their roles. Consequently, several participants felt empowered to carve out their own space and engage in hobbies without experiencing guilt [4]. Seeking assistance from fellow caregivers or professionals also assisted them in regaining control over their circumstances [7]. Furthermore, the program provided caregivers with the tools to identify and establish their boundaries. By asserting their boundaries, they learned to maintain control over their own lives [3].

Meditation training enhanced participants’ ability to adapt to stimuli, promoting flexibility in their responses [6]. By focusing on breathing, caregivers develop greater self-awareness and calmness [6]. Structured exercises encouraged reflection on current situations and desired changes, facilitating active self-evaluation [3].

Within the realm of personal growth, the program facilitated caregivers in actively considering their decision-making processes, enabling them to delineate their priorities [3]. Caregivers developed a heightened awareness of their actions and reflected on their circumstances through the support program [3]. Caregiving for individuals with ALS presented unexpected opportunities for personal growth. Participants found themselves reevaluating their lives and priorities, experiencing strengthened familial bonds, and acknowledging the transient nature of their caregiving roles [4]. This newfound perspective led to a deeper appreciation for their caregiving roles and a recognition of their capacity for growth in unforeseen circumstances [4].

- Instrumental contribution

This sub-theme primarily focuses on the instrumental contributions of psychosocial interventions for family members of individuals with ALS. It explores how these interventions provide information, practical tools, and support to help family members cope with the psychosocial challenges associated with the disease.

The intervention equipped caregivers with valuable and trustworthy information, offering practical tools to navigate future challenges, emotions, and negative thoughts. By gaining fresh insights and learning from the experiences of fellow caregivers, they developed a deeper understanding and personal perspective [3,13]. Consequently, caregivers reported enhanced confidence in their ability to face the future and embraced the inherent challenges with greater acceptance. Some professionals explained important issues related to the disease and its progressions even if the diagnosis is different [3,15]. The participants better understood their situations and challenges and how to handle them. The meeting provided them with new ideas on how to approach current or future challenges [13].

One of the reasons for caregivers of people with ALS to participate in interventions was to prepare for the later stages through other caregivers’ advice [4,13,15]. The less experienced participants embraced the lived experiences of the more experienced participants by expressing the importance of and their appreciation for learning from peers, which they found useful as preparation for the difficult time ahead of them. The desire to help other members caused them to share their personal stories to prepare these members for the future [13]. The intervention also allows a space where one can consult and develops the individual’s ability to ask for help [3].

### 3.4. Theme 2: Interpersonal Benefits

This theme includes the individual feeling of the participants within the group along with the effect of the intervention on interpersonal relationships between the participants and improvement of the relationship in the martial aspect.

Intervention meetings were characterized by a special atmosphere, acceptance, consideration, mutual understanding, and sympathy for each participant without being judged [13]. Although the participants saw sharing as a resource [4], they described having no one else to share their thoughts and emotions with, because they did not expect people without personal experience to understand their situation [13]. They liked having a personal space in which to have the possibility to receive support and vent and share feelings, emotions, and experiences with people undergoing similar experiences [3,4,6,13,15].

Participants expressed a positive perception of their involvement in the group despite the challenges of their busy lives, emphasizing the significance of the meetings. They felt a sense of belonging to a community where mutual sympathy for everyday challenges was emphasized. Despite their diverse backgrounds and circumstances, the shared experience of caregiving for ALS patients facilitated understanding, connection, and support among group members. Participants described the evolution of relationships within the group and highlighted the meaningfulness of peer support [13].

In the online intervention, caregivers utilized private messaging and shared tips and advice, fostering connections with one another. Direct messaging proved invaluable as it enabled the exchange of personal stories and experiences [3]. Following the conclusion of the group sessions, adult children participants felt apprehensive but established a WhatsApp group to stay connected [4]. Conversely, partners of individuals with ALS did not maintain communication post-group completion [4].

In the martial aspect, the program increased participants’ awareness of their relationship with their partner, prompting some caregivers to make changes. It improved their understanding of their partner’s needs and emotions, encouraging them to communicate more effectively about difficult topics. This improved communication strengthened their relationship [3].

### 3.5. Theme 3: Charting Challenges and Pathways to Improve Psychosocial Interventions

This theme addresses barriers and points for improvement in the interventions. It includes three sub-themes: (1) Heterogeneity in group composition, (2) Focusing on bureaucratic aspects, and (3) The added burden of participating in the intervention.

- Heterogeneity in the group

Difference between adult children of people with ALS and parents who care for a child with ALS that are joint in the same group in an intervention may presented challenges [4]. Likewise, participating in a group where the levels of disease progression are different may raise barriers to sharing in the group: for example, caregivers whose partner is in an early stage felt that no questions have yet been raised or that it would be too confrontational to talk to a peer caring for a person with ALS in a more advanced stage [3]. Also, the joint participation of people with ALS and caregivers is another reason for the difficulty in participating in a group [15].

- Focusing on bureaucratic aspects

Excessive focus was directed toward criticizing the healthcare system, overshadowing discussions within the partners’ group. Main topics predominantly revolved around technical aspects, including caregiving strategies and available services. These discussions encompassed practical and bureaucratic care elements alongside caregivers’ emotional well-being and concerns [4]. Participant observations revealed instances where discussions focused on the affected relative rather than the challenges faced by the caregivers themselves [13].

- The added burden of participating in the intervention

A few caregivers expressed feeling overwhelmed by the program, while others found themselves consumed by caregiving duties, leaving little time for other pursuits. However, some participants felt the intervention was not beneficial, as they had not felt the need for support yet. Instead, they preferred to allocate their time to other activities rather than focusing on the disease [3]. Participants frequently reported experiencing distress following group meetings [15]. Many described initial feelings of tension or stomach discomfort before and during the first meeting, which gradually gave way to sensations of relief, peace, gratitude, and reduced feelings of loneliness and frustration [13]. Notably, in De Wit et al.’s [7] study, the integrated psychosocial support program did not result in a reduction in psychological distress or caregiver burden nor did it improve the quality of life for caregivers or patients.

## 4. Discussion

The purpose of the study is to review the existing knowledge on psychosocial interventions for family members caring for people with ALS. The knowledge mapping will help identify gaps according to which it will be possible to summarize recommendations for future studies and implications for practice that will help improve the well-being and lives of the caregivers. There are many studies on the burden of caregivers of people with ALS in general, but there is not much knowledge about interventions for caregivers. Even in the existing literature on interventions in the field of ALS, the focus is mainly on the person with ALS and not on caregivers.

Three major themes emerged from the data: (1) Personal benefits; (2) Interpersonal benefits; and (3) Charting challenges and pathways to improve psychosocial interventions. The findings can be viewed and understood considering the resource conservation theory [16], which offers a comprehensive framework for understanding the impact of evaluation on emotions and performance by focusing on the resources of individuals and groups. According to the theory in stressful situations, a process of resource loss occurs, but alongside, it a process of resource gain occurs. The main motivation of humans according to the theory is to build, protect, and nurture their reserves of resources to protect the self and the social relationships that support the self. The key to maintaining mental health positively includes successfully preventing resource depletion, accumulating existing resources, or adding new resources [17,18]. This can be seen in regard to the psychological benefits gained by caregivers participating in psychosocial interventions. Caregivers experienced benefits such as feelings that they were being acknowledged, taking a moment for oneself, gaining a sense of control and confidence, and fostering personal growth. Likewise, in regard to instrumental contributions of the interventions, caregivers gained resources such as information practical tools. Additionally, in regard to interpersonal benefits, caregivers enhanced their interpersonal relationships both socially and in the marital aspect.

Furthermore, it seems that the various contributions of psychosocial interventions for caregivers mitigate the process of loss spirals, which refers to how the loss of one resource can lead to the loss of additional resources [19], while also gaining new resources.

While the review uncovered psychological, instrumental, and interpersonal benefits for psychosocial interventions for caregivers, it is essential to note the findings of Sharbafshaaer et al. [14]. In this study, despite the implementation of psychological support via telemedicine during the COVID-19 pandemic, it was found that the short-lasting intervention did not yield significant differences in reducing caregivers’ burden or perceived stress nor did it enhance their resilience. This underscores the complexity of providing effective support to caregivers, especially in challenging circumstances such as a global health crisis.

## 5. Limitations

The search process included only studies published in English. The review includes only peer-reviewed articles published as of now, additional unpublished studies may have been conducted. Although two researchers were evaluated, which indeed strengthens the validity of the test, but things may have been missed due to human error.

## 6. Salient Gaps in the Literature and Implications for Future Research

A limited number of studies and limited samples of family members who care for people with ALS participated in the studies. Further research should include additional interventions as well as increased sample size. There is a clear gender gap and a lack of knowledge about male caregivers. Furthermore, there is also a lack of research on caregiving children; more research is needed among these groups. The studies only took place in three countries, Italy, Denmark, and the Netherlands. and this does not reflect the situation in the rest of the countries in the world. Specifically, there is a lack of research on traditional populations; all the studies that have been published refer to Western populations. Thus, there is a need to expand the research in other countries, especially in traditional countries and areas that have not yet been explored. In addition, the review presents preliminary evidence for interventions that combine body and mind, but the field is not sufficiently developed; therefore, it is important to develop such interventions.

## 7. Practical Implications

Based on the findings in the current study, guidelines for interventions are presented. Since the psychoeducational component is central in these interventions, the importance of the role of the facilitator is emphasized. The facilitators of these interventions must have knowledge, expertise, and experience in the ALS and caregiving fields. Facilitators must get to know the different stages of the disease, the different struggles in these stages, and the specific challenges of each stage. The findings show that there is a tendency to put the focus on technical content and anger toward the health system, and it is important that the facilitator guides the group to focus on the emotional aspects so that on the one hand, he will give it a place, but it will not be the main thing. A facilitator needs to have experience in the field because this gives the facilitator a better understanding of the problems they may encounter as caregivers and the caregivers a feeling that they are understood [3].

It is important to separate the groups in terms of the characteristics of the participants, such as caregivers and people with ALS separately, family closeness to the people with ALS, and stages of the disease, because the needs, the coping, and the contents that arise in the sessions are different.

In interventions, many times there are exercises, and it is important to provide feedback on exercises within the program as it helps caregivers gain insights into their thoughts and feelings, and it even motivates them to continue with the intervention. Also, giving feedback will help reflect the caregivers’ situation, and they will be offered advice. The feedback also confirms the validity of the feelings and actions and will help caregivers feel understood and encouraged. It is very important to adapt the feedback to the caregivers’ personal situations to make them feel that they are being listened to and taken seriously.

It is important to develop online interventions so that even if caregivers are geographically distant, they can take part in the most suiting interventions for them. At the same time, it is important to adjust the response according to the stages of the disease, the needs that arise, and the nature of the program. In addition, because not all caregivers know how to use online programs efficiently due to technological difficulties, or may prefer to take part in an intervention not online, it is important to continue providing face-to-face interventions.

It is important to consider that integrating into the group takes time, and it is possible that some will integrate at first and others will take longer to integrate, and they may even experience a variety of emotions. Therefore, it is important to show sensitivity toward the participants. Also, it is important to allow the participants flexibility and not burden them with tasks that will add to their caregiver burden.

## 8. Conclusions

There is a lot of research that addresses negative aspects of caregiving, including the caregivers’ burden, their needs, and the impact on their well-being, but there is limited research on the psychosocial interventions for them. It is evident that the literature tends to focus on loss and challenges and does not give enough attention to factors that promote resilience. it is also important to deal with aspects that focus on resilience and the development of adapted and sensitive interventions that will meet the needs and allow the caregivers to deal with the crisis that befell them and grow from it. The present review summarized trends in research on psychosocial interventions and found that these interventions have multidimensional effects.

At the individual level, the intervention provided knowledge and tools that improve the mental and emotional state of the participants, their well-being, and their sense of control and confidence, and it allows them to take a moment for themselves. The intervention even provides them with a feeling of acknowledgment and contributes to personal growth.

In the social dimension, the intervention is a resource that strengthens the resilience of the participants by sharing knowledge and tools that help them cope and strengthen the relationships between them as a group and each of them with their family members, the self-confidence, the feeling of belonging to the community, and that they are not alone.

Based on the findings, practical guidelines were formulated that focus on the group’s composition, the facilitator’s role, the contents, the relationships within the group, and the opportunities and limitations of online interventions.

## Data Availability

Data sharing is not applicable.
